# Intervention Effects of Ganoderma Lucidum Spores on Epileptiform Discharge Hippocampal Neurons and Expression of Neurotrophin-4 and N-Cadherin

**DOI:** 10.1371/journal.pone.0061687

**Published:** 2013-04-24

**Authors:** Shu-Qiu Wang, Xiao-Jie Li, Shaobo Zhou, Di-Xiang Sun, Hui Wang, Peng-Fei Cheng, Xiao-Ru Ma, Lei Liu, Jun-Xing Liu, Fang-Fang Wang, Yan-Feng Liang, Jia-Mei Wu

**Affiliations:** 1 Department of Pathophysiology, School of Basic Medical Sciences, Jiamusi University, Jiamusi, Heilongjiang Province, P. R. China; 2 School of Rehabilitation Medical Sciences, Jiamusi University, Jiamusi, Heilongjiang Province, P. R. China; 3 Children Neural Rehabilitation Laboratory of Jiamusi University, Jiamusi, Heilongjiang Province, P. R. China; 4 Department of Life Science, Institute of Biomedical and Environmental Science and Technology, University of Bedfordshire, Luton, United Kingdom; Oregon Health & Science University, United States of America

## Abstract

Epilepsy can cause cerebral transient dysfunctions. Ganoderma lucidum spores (GLS), a traditional Chinese medicinal herb, has shown some antiepileptic effects in our previous studies. This was the first study of the effects of GLS on cultured primary hippocampal neurons, treated with Mg^2+^ free medium. This *in vitro* model of epileptiform discharge hippocampal neurons allowed us to investigate the anti-epileptic effects and mechanism of GLS activity. Primary hippocampal neurons from <1 day old rats were cultured and their morphologies observed under fluorescence microscope. Neurons were confirmed by immunofluorescent staining of neuron specific enolase (NSE). Sterile method for GLS generation was investigated and serial dilutions of GLS were used to test the maximum non-toxic concentration of GLS on hippocampal neurons. The optimized concentration of GLS of 0.122 mg/ml was identified and used for subsequent analysis. Using the *in vitro* model, hippocampal neurons were divided into 4 groups for subsequent treatment i) control, ii) model (incubated with Mg^2+^ free medium for 3 hours), iii) GLS group I (incubated with Mg^2+^ free medium containing GLS for 3 hours and replaced with normal medium and incubated for 6 hours) and iv) GLS group II (neurons incubated with Mg^2+^ free medium for 3 hours then replaced with a normal medium containing GLS for 6 hours). Neurotrophin-4 and N-Cadherin protein expression were detected using Western blot. The results showed that the number of normal hippocampal neurons increased and the morphologies of hippocampal neurons were well preserved after GLS treatment. Furthermore, the expression of neurotrophin-4 was significantly increased while the expression of N-Cadherin was decreased in the GLS treated group compared with the model group. This data indicates that GLS may protect hippocampal neurons by promoting neurotrophin-4 expression and inhibiting N-Cadherin expression.

## Introduction

Epilepsy, a condition caused by abnormal, disorderly discharging of cerebral neurons, can cause transient dysfunctions of the brain [1], [2]. Epilepsy is usually controlled, but cannot be cured with drugs, although surgery may be considered in difficult cases. The fungus Ganoderma lucidum has been used for centuries in East Asia. Its fruiting body is called “Lingzhi” in China. Ganoderma lucidum spores (GLS) are ejected from the pileus, of growing Ganoderma, in the mature phase of the fungus’ growth. GLS contains many ingredients, including triterpenoids, polysaccharide, amino acids, polypeptides, sterols, alkaloids, fatty acids, vitamins and inorganic ions [3]. The diversity of bio-active ingredients in GLS is associated with the universality of its multiple pharmacological effects. Ganoderma lucidum has been a popular folk medicine to treat various human diseases, such as hepatitis, hypertension, hypercholesterolemia and cancer [4]–[6].

The effect of GLS treatment in epileptic animal models has been tested in our laboratory and the results revealed that GLS reduced the apoptosis of nerve cells caused by epilepsy, suppressed the expression of NF-κB, facilitated the immune reactivity of IGF-1 [7] and reduced the levels of IL-6 in the brain [8]. However, more research is needed to explore the anti-epileptic effects of GLS as there have been no studies on neuronal morphology during GLS treatment. Furthermore, there are no reports on the effects of GLS on the expression of Neurotrophin-4 (NT-4) or N-Cadherin, proteins that are expressed in hippocampus neurons and known to play an important role in neuron development [9]–[13].

NT-4 consists of 130 amino acids and is a member of the neurotrophin (NT) family. NT-4 is expressed widely in the brain, it is mainly expressed in the hippocampus [9]. NT-4 generates a variety of biological effects through combining with receptors in the membranes [10], [11]. Long-term use of NT-4 has been shown to inhibit hippocampal neuronal death by up to 50% [12]. Neurotrophin can also mediate neuroprotection of hippocampal neurons following traumatic brain injury [12], [28]. N-Cadherin is a member of the cadherin family with many biological activities. Cadherin plays an important role in targeting the growth of axons and the construction of correct synaptic connections [13]. GLS has also been shown to have a neuroprotective effect, however, no studies have been performed to detect the influence of GLS on the expression of NT-4 or N-Cadherin in hippocampal neurons.

This study investigated the anti-epileptic effect and mechanism of GLS activity using primary hippocampal neurons from rat brain in an *in vitro* model [14]. The effects of GLS on neuron morphology and purity, during the culture period, were assessed and the purity of hippocampal neurons was measured in order to determine the best culture time. Hippocampal neurons were confirmed by immunofluorescent staining of neuron specific enolase (NSE). In addition, the sterile method for GLS generation was investigated in order to fully ascertain the requirements of the neuron culture, and the maximum non-toxic concentration of GLS. The optimal concentration of GLS to use with this model was also determined. Finally, the expression of NT-4 and N-Cadherin in neurons of GLS and control treated cells were analysed in order to determine any correlation of anti-epilepsy effect and a potential mechanism of action for GLS.

The results showed that GLS treatment increased the number of normal hippocampal neurons and preserved the morphologies of hippocampal neurons well. Furthermore, the expression of NT-4 was significantly increased while the expression of N-Cadherin was decreased compared with the model group. These data indicate that GLS may protect hippocampal neurons by promoting NT-4 expression and inhibiting N-Cadherin expression.

## Materials and Methods

### Animals

Newborn Wistar rats (up to 24 hours old) were purchased from the Animal Center of Jiamusi University. Animal study was performed under the guidelines of the animal ethical committee in JiaMusi University.

### Cell Culture and Culture Medium of Hippocampal Neurons

Hippocampal tissues from rats were harvested using conventional methods [15]–[17] and a 5×10^4^/ml cell suspension was made. 6 ml cell suspension was transferred into 100 ml culture flasks; 0.2 ml cell suspension was transferred into each well of a 96-well plate; and 1 ml cell suspension was transferred into each well of a 24-well plate respectively. Cultures were incubated in a 37°C incubator (containing 5% CO_2_) for 24 hours, after which time the whole cultured medium [Neurobasal medium (Cat. No. 21103049, Gibco), 2% B27 supplement (Cat. No. 17504044, Gibco), 0.5 mmol/L glutamine and 10% FBS] was replaced by the nutrient maintaining medium [Neurobasal medium, 2% B27 supplement and 0.5 mmol/L glutamine]. Half amount of the volume of medium was changed every other day.

### Identification of Hippocampal Neurons

Hippocampal neurons were identified by detection of NSE which is highly expressed in neurons. Immunofluorescence technique was used. Briefly, neurons were cultured for 9 days, the medium was removed and the neurons were rinsed 3 times using 0.01 mol PBS for 5 minutes each time. The hippocampal neurons were then incubated with anti-NSE antibody (Cat. No BA0535, Wuhan Boster Bio-Engineering Ltd, China) and then incubated with secondary antibody labeled with FITC (Cat. No. BA1105, Wuhan Boster Bio-Engineering Ltd, China). Pictures were taken under the fluorescent microscope.

### Evaluation of the Purity of Hippocampal Neurons

Hippocampal neurons were cultured to a density of 2.5×10^4^/ml. Four culture flasks were used respectively for day 5, 7, 11 and 14. The number of hippocampal neurons and glial cells were counted in 10 randomly selected visual fields under a 100× microscope. The purity of hippocampal neurons was calculated by using the following formula: the number of hippocampal neurons/(the number of hippocampal neurons+the number of glial cells).

### Establishment of the Epileptiform Discharge Hippocampal Neuron Model

The epileptic cell model was set up using a conventional method [5]–[8]. The nutrient medium of hippocampal neurons, cultured for 9 days, was changed into magnesium ion (Mg^2+^) free extracellular medium (medium (145 mmol NaCl, 2.5 mmol KCl, 2 mmol CaCl_2_, 10 mmol HEPES, 10 mmol glucose, 0.002 mmol glycine, pH 7.2, 290+10 mOsm) and treated for 3 hours. This induced permanently manifested recurrent, spontaneous seizure discharges characteristic of the same electrographic properties seen in human epilepsy [14]. Following treatment, this medium was replaced with the normal culture medium (145 mmol NaCl, 2.5 mmol KCl, 2 mmol CaCl_2_, 1 mmol MgCl_2,_ 10 mmol HEPES, 10 mmol glucose, 0.002 mmol glycine, pH 7.2, 290+10 mOsm) and incubated for a further 6 hours.

### Sterile Treatment and Set up for Appropriate Concentration of GLS

GLS were processed by low-temperature intermittent sterilization. 1.25 g of GLS were weighed and put in a 10 ml centrifuge tube which was then sealed with sterile adhesive tape. The tube was put in an electrothermal constant-temperature dry box at 70°C for 1 hour and then the tube was put at room temperature for 24 hours. The above procedures were repeated in a sterile environment within 2 weeks. Different quantities of sterilized GLS were added into culture flasks and the flasks incubated at 37°C. Observations were undertaken under microscope at the 6^th^, 12^th^, 24^th^, 48^th^ and 72^nd^ hour respectively.

### Determination of Maximum Non-Toxic Concentration of GLS to Hippocampal Neurons

A 0.125 g/ml suspension was prepared by adding GLS into a maintaining nutrient medium and the solutions were diluted into 12 different concentrations (1∶256, 1∶512, 1∶1024, ……). 0.1 ml of each concentration was put into 8 wells of a 96-well plate containing 0.2 ml hippocampal neurons (5×10^4^/ml cell suspension) cultured for 9 days. In the meantime, a control group with normal cells was also set up. The 96-well plates were incubated in a CO_2_ incubator at 37°C. Any changes in cell morphology were recorded on day 3. The experiment was repeated three times.

### Determination of an Optimized Concentration of GLS

A 24-well plate of hippocampal neurons (as detailed in section 2.2) cultured for 9 days was used for this assessment. They were randomly divided into the following groups:

- Normal control group: the medium was changed into normal medium and “treated” for 3 hours, then replaced with normal medium and cultured for a further 6 hours;
- Model of epileptiform discharge hippocampal neurons group *(*named as model group): the medium was changed into a medium that had the same components as the normal medium except it was magnesium free. Cells were treated with this for 3 hours, then this culture medium was replaced with normal culture medium and cells cultured for a further 6 hours;

GLS group: based on the experiment in section 2.6. the concentration of GLS was divided into low, medium and high, (0.061 mg/ml, 0.122 mg/ml and 0.224 mg/ml) respectively. GLS in a magnesium free culture medium was used to culture the hippocampal neurons for 3 hours, then replaced with a normal culture medium and the cells were cultured for a further 6 hours. iii) In GLS group I, the culture medium was replaced with a magnesium ion free medium containing 0.122 mg/ml of GLS for 3 hours, and then replaced with a normal culture medium and cells were cultured further for 6 hours. iv) In GLS group II, the culture medium was replaced with a magnesium ion free medium for 3 hours, and then a normal culture medium containing 0.122 mg/ml of GLS replaced this and neurons were cultured for a further 6 hours.

The hippocampal neurons were labeled using conventional immunofluorescent techniques as stated above. Images were taken under the fluorescence microscope. Fifteen neurons were chosen randomly from each group and the absolute value of fluorescence intensity was assayed using Image-Pro Plus 6.0 software [18], [19].

### Western-blot Analysis of NT-4 and N-Cadherin Expression

Proteins were prepared using commercial lysis buffer (Cat.No. is P0013 Beyotine Institute Biotechnology, China) according to the product protocols. Protein sample was mixed with 5× loading buffer solution, denatured for 5 minutes at 100°C, and then subjected to 12% sodium dodecylsulfate-polyacrylamide gel electrophoretic separation followed by transferal of the protein to a nitrocellulose filter. The nitrocellulose filter was blocked with 1% bovine serum albumin overnight. It was then incubated with a rabbit-anti-β-actin polyclonal antibody (BAB) rabbit-anti-NT-4 BAB and rabbit-anti-N-Cadherin BAB (Cat. No. is BA2305, BA1294, BA0673 respectively, Wuhan Boster Bio-Engineering Ltd, China) respectively for 2 hours at 37°C, rinsed with Tris-buffered saline with Tween 20 (TBST) 3 times for 10 minutes each, and then incubated with horse radish peroxidase-labeled goat-anti-rabbit IgG antibody for 1 hour at 37°C. The blot was then rinsed twice for 10 minutes with TBST solution, rinsed once for 10 minutes with TBS solution again, and finally stained using 5-bromo-4-chloro-3-indolyl phosphate/nitro blue tetrazolium.

### Statistical Analysis

Data were shown as mean ± standard deviation and the results were analyzed using Microsoft Excel 2007 and SPSS13.0. There was significant statistical difference when *P*<0.05.

## Results

### Observation of Hippocampal Neuron Morphology

**Figure 1:**
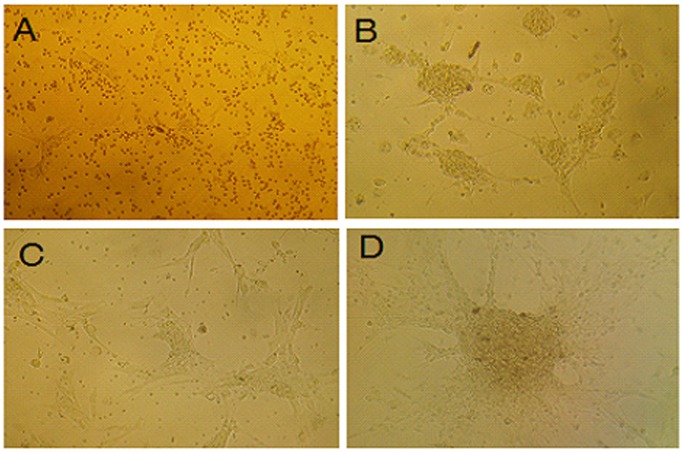
Hippocampal neurons (x100). (**A**) Cultured at 24 hours, part of the cells stretched out neurites with different thickness, and the somas were cone or polygon-shape; (**B**) cultured at day 3, the neurons increased and the neurites connected into a network; (**C**) cultured at day 5, the haloes of the neurons were obvious, the volume of the somas became bigger, the neurites were dense, thick and long; (**D**) cultured at day 9, the neurons became mature and aggregated into clumps, the whole growth of the neurons were unevenly distributed, and it was difficult to identify a single neuron.

Initially upon harvesting, the hippocampal neurons were round or oval, transparent, single or several cells in a cluster, and evenly distributed. After 24 hours, the cells had adhered, some of the cells stretched out neurites with different thickness, and the somas were cone or polygon-shape **(** **Figure 1** **-A);** After 3 days, the number of the neurons increased and the neurites connected into a network **(** **Figure 1** **-B);** 5 days later, the haloes of the neurons were obvious, the volume of the somas became bigger, the neurites were dense, thick and long **(** **Figure 1** **-C);** At day 9, the neurons became mature and aggregated into clumps, the whole growth of the neurons were unevenly distributed, and it was difficult to identify a single neuron **(** **Figure 1** **-D).**

### Identification of the Hippocampal Neurons by NSE

**Figure 2:**
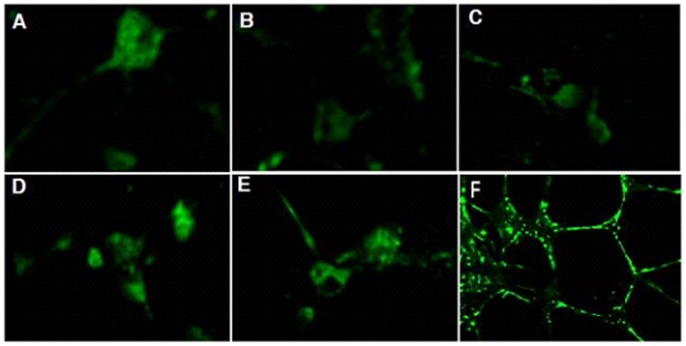
Expression of NT-4. NT-4 expression in control group **(** **Figure 2** **-A)**, model group **(** **Figure 2** **-B)**, low concentration of GLS group **(** **Figure 2** **-C)**, culture medium concentration of GLS group **(** **Figure 2** **-D)**, high concentration of GLS group **(** **Figure 2** **-E)**. immunofluorescent labeling of the hippocampal neurons, the cytoplasm and the neurites were green, while the nuclei were stainless **(** **Figure 2** **-F)**.

The results from NSE immunofluorescent staining showed that the somas of the hippocampal neurons were plump, triangular, round, fusiform or polygonal; the neurites were thick and interweaved into a network; the cytoplasm and the neurites were green revealing the presence of NSE, while the nuclei were stainless **(** **Figure 2** **-F)**.

### The Purity of Hippocampal Neurons

The hippocampal neurons cultured under low-density conditions were observed under the microscope. The hippocampal neurons did not differentiate completely until day 9. The somas of the neurons were plump, round, fusiform, triangular or polygonal and the neurites were dense, thick and interweaved into a network. It was rare to see glial cells and the purity of the neurons was up to 96±2.5%. At day 11 and 14, the hippocampal neurons grew well compared with day 9 and the purity of the neurons had not obviously changed **(** **Table 1** **).**

**Table 1:** The purity of the hippocampal neurons.

Time (day)	5	7	9	11	14
Hippocampal neurons	33.1±11.18	63.0±17.73	83.1±23.85	67.0±18.81	80.9±17.78
Glial cells	4.5±2.07	5.1±1.66	3.0±1.41	2.7±1.34	3.0±1.15
Purity (%)	0.876±0.046	0.918±0.032	0.960±0.025	0.964±0.010	0.963±0.018

### The Maximum Non-Toxic Concentration of GLS on Hippocampal Neurons

The toxic effects of GLS on the hippocampal neurons induced an increase in granules in the cytoplasm, a decline in the transparency, cracking of the neurites, deforming and detachment of the cells. With the decline of GLS concentration, the toxic effects gradually weakened and the survival rate of the neurons increased. According to the pharmaceutical dilution, the maximum non-toxic concentration of GLS was calculated as 0.122 mg/ml.

### Identification of the Optimal Concentration of GLS

**Figure 3:**
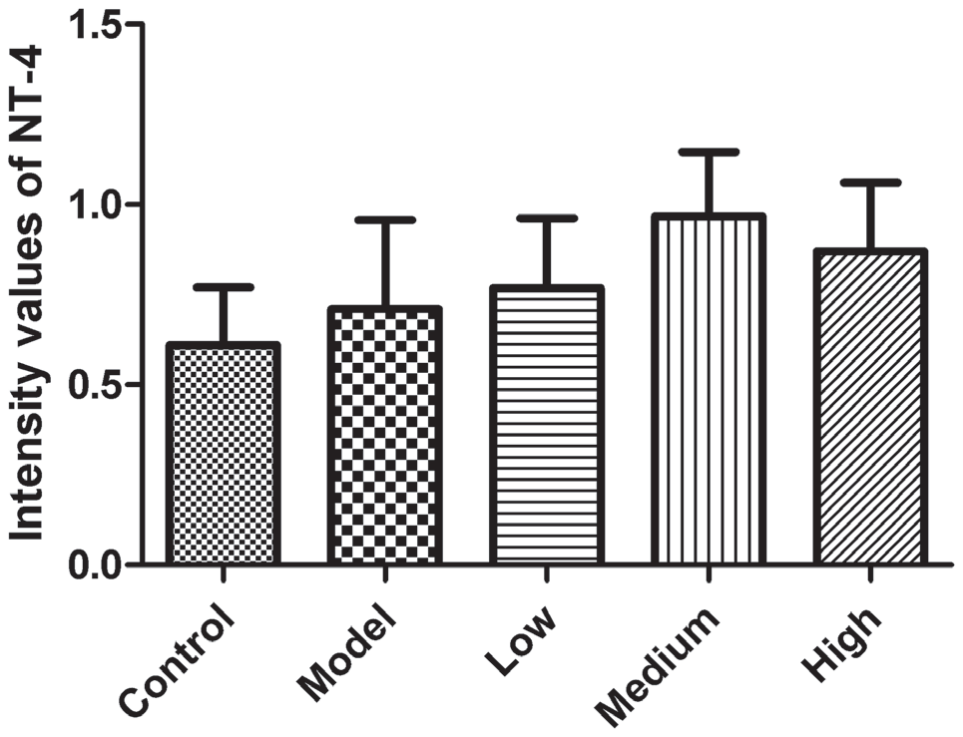
Fluorescent intensity values of NT-4 in different groups. There is a significant increase in the expression of NT-4 in the model group compared to the control group (p<0.01); the expression of NT-4 at each concentration (low, medium and high) of GLS group was higher compared with either the model group or normal groups (p<0.01), the differences among all the groups were statistically significant (p<0.05).

The results from immunofluorescent staining of NT-4 revealed that NT-4 was mainly located in the cytoplasm **(** **Figure 2** **A–E)**, and the expression of NT-4 in the model group was elevated compared with that in the normal control group (p<0.01); the expression of NT-4 in each concentration of GLS group was high compared with the model group (p<0.01), the differences among all the groups were statistically significant (p<0.05), and the expression of NT-4 at the 0.122 mg/ml concentration of GLS was the highest **(** **Figure 3** **).**

### Western-blot Analysis of NT-4 and N-Cadherin

**Figure 4:**
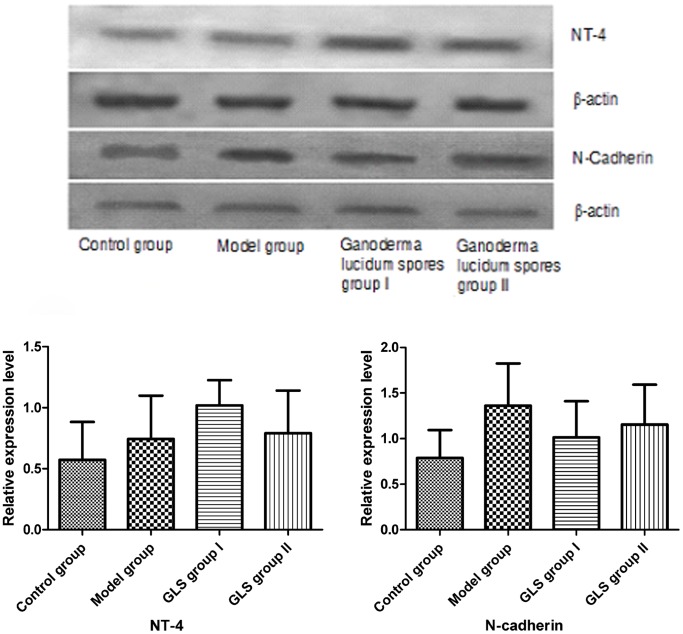
The expression level of NT-4 and N-Cadherin in different groups. Compared with the model group, the expression of NT-4 was higher in the GLS group I (GLS dissolved into Mg ion free medium and incubated for 3 hours) and II (neurons incubated with Mg ion free medium for 3 hours then incubated with GLS dissolved into the normal medium for 6 hours), and the expression of N-Cadherin was lower. Compared with the control group, the expression of NT-4 and N-Cadherin were higher in GLS group I and II.

The results from the Western-blot assay showed that NT-4 and N-Cadherin were expressed in neurons from all groups **(** **Figure 4** **)**. In the GLS group I, the expression of NT-4 was higher compared with the model group (p<0.01), and also higher than the GLS group II. In comparison, the expression of N-Cadherin in neurons from Group I was lower compared with the model group (p<0.01), but more elevated than that seen in the control group (p<0.01) and there was no significant difference in N-Cadherin expression between GLS group I and II. In GLS group II, the expression of NT-4 was higher compared to the model group (p<0.05), while the expression of N-Cadherin was lower compared with the model group (p<0.05), but higher than observed in the control group (p<0.05) **(** **Figure 4** **)**.

## Discussion

Currently, there are no specific drugs with few side effects to cure epilepsy. Improper use of anti-epilepsy drugs by epilepsy patients can result in severe consequences including disability and mortality. Therefore, development of highly-efficient anti-epilepsy drugs with less side-effects has become a great challenge to the life science researcher. GLS, the germ cells of Ganoderma, have shown many pharmacological effects with low toxicity in animal models over the last 10 years [3], [7]–[9], [20], [21]. However, there have been few studies on the anti-epilepsy effects of GLS and no studies on neuron morphology during GLS treatment. Furthermore, there are no reports on the effects of GLS on the expression of NT-4 or N-Cadherin. Therefore, the present study was performed using epileptic neurons, a model used widely to screen anti-epileptic drugs *in vitro*, in order to investigate the anti-epilepsy effect of GLS.

The morphology of hippocampal neurons, at different culture time points, was used to establish the optimal conditions for the culture of neurons. At day 9, the neurons became mature and aggregated into clumps and the whole growth of the neurons was unevenly distributed **(** **Figure 1** **-D).** The results from NSE immunofluorescent staining **(** **Figure 2** **-F)** confirmed the successful culture of hippocampal neurons with a purity of 96% at day 9 until day 14, thus, neurons cultured at day 9 were chosen for subsequent analysis. The maximum non-toxic concentration of GLS on hippocampal neurons was 0.122 mg/ml (this concentration did not increase granules in the cytoplasm or elicit a decline in the cell transparency). This 0.122 mg/ml GLS concentration, and its ½ or 2X concentration were used to evaluate NT-4 expression in neurons cultured under the different treatment regimens.

The results showed that NT-4 is mainly localised in the cytoplasm **(** **Figure 2** **A–E)**, and that the expression of NT-4 in the model group was higher than that seen in the normal control group (p<0.01). The expression of NT-4 in neurons from each of the GLS groups was higher compared to the model group (p<0.01). The differences observed in the groups were statistically significant (p<0.05), with the expression of NT-4 in the group containing 0.122 mg/ml GLS being the greatest **(** **Figure 3** **).** Results also demonstrated that GLS promoted the expression of NT-4 regardless of whether it was added into the Mg^2+^ free culture medium for 3 hours (GLS group I) or 3 hours after the Mg^2+^ free culture medium incubation was replaced with GLS dissolved in normal culture medium for 6 hours (GLS Group II) (**Figure 4**). However, the NT-4 expression in GLS group II was significantly decreased compared to that of GLS group I. This can be explained by the different culture conditions in the model providing a possible mechanism of activity for GLS, where the GLS dissolved in the Mg^2+^ free culture medium for 3 hours seems to prevent the side effects caused by the loss of Mg ^2+^,or delay its effects. When GLS was given after the epileptic model was set up, i.e. after the 3 hours treatment with Mg^2+^ free culture medium, it was observed that GLS action could limit the damage but the effects were weaker compared to when GLS was present in the Mg^2+^ free medium. Results also showed that GLS inhibited the expression of N-Cadherin induced by Mg^2+^ free culture medium incubation (**Figure** 4), under Group I or Group II conditions.

NT-4 expression can protect neurons from apoptosis caused by low potassium [22]. An *in vitro* experiment demonstrated that the functions of NT-4 in the central nervous system could support the expression and survival of hippocampal cholinergic neurons, noradrenergic neurons, striatal GABA-ergic neurons and dopaminergic neurons during the embryonic period [23]–[27]. NT-4 can protect the neurons of embryonic rats from injury caused by a lack of glucose [28]. In NT-4 rich culture mediums, neurons possess a strong ability to resist toxicity induced by calcium ion carriers A23187m, demonstrating the importance of NT-4 in this process of protection from injury induced by calcium. NT-4/5 was shown to support the survival of cholinergic neurons cultured *in vitro*, and increased the expression of choline acetyltransferase in these cells. NT-4 can produce a NGF-like protecting effect on cholinergic neurons [29]. NT-4 can also adjust the plasticity of neurons, promote neuromuscular junction formation and induce the normal motor neurons to sprout lateral branches [30]. Engelhardt *et al*. found that retinal ganglion cells treated with sub-toxic concentrations of glutamic acid, N-methyl-D-aspartic acid, kainic acid (KA) and guinolinic acid induce the expression of brain-derived neurotrophic factor and NT-4, facilitating resistance to toxicity of high concentrations of glutamic acid [31]. NT-4 can promote neuronal survival, growth and differentiation by protecting hippocampus and cortical neurons allowing them to resist exitotoxins and metabolic injuries [32].

Epileptic seizures are associated with mossy fiber sprouting of hippocampal neurons, synaptic reconstructions, cell apoptosis and excitatory neurotransmitters. After epileptic seizures, there is not only hypoxia and ischemia of brain tissues, but also the loss of neurons and many complicated pathological changes. It has been shown that, post-epileptic pathological changes of the brain mainly involve the loss of neurons, the proliferation of glial cells [33], [34]. Because NT-4 can promote neuron survival, alleviate neuronal injuries, inhibit neurons from apoptosis and adjust the synapses plasticity, NT-4 might have an anti-epileptic seizure role.

Postictal mossy fiber sprouting of hippocampal neurons and synaptic reconstructions are important in the pathogenesis of epilepsy and are prevalent phenomena of the condition. Neural circuits formed by mossy fiber sprouting could increase granular cell excitability, which is closely related to the generation and spread of epileptiform discharge [35]–[38]. Dudek *et al*. found that the mossy fiber sprouting participated in the formation of new excitatory circuits in the dentate gyrus [39]. Fujita *et al*. discovered that after a KA-induced convulsive model was injected with KA for 12–24 hours, the mRNA expression of N-Cadherin decreased with a reduction of neurons, while from 48 hour to day 7 the mRNA expression partly recovered. However, after exposure to KA for 48 hours, N-Cadherin expression significantly increased in surviving neurons [40].

In conclusion, this study has utilised primary cultured neurons to test the optimum concentration of GLS along with the culture time for the hippocampal neuron in order to assess the effects of GLS on the morphology of hippocampal neurons and the expression of both NT-4 and N-Cadherin when GLS is dissolved into Mg^2+^ free medium directly or into normal medium following epileptic neuron model set up. The results showed that GLS may indirectly inhibit mossy fibers sprouting and adjust the synaptic reconstructions by inhibiting the expression of N-Cadherin, which may inhibit the neural circuit formed by mossy fiber sprouting providing a mechanism for its anti-epileptic effects. By promoting NT-4 expression, GLS can elicit a protective effect on hippocampal neurons by promoting neuronal survival and alleviating the injuries of postictal hippocampal neurons thereby enhancing its anti-epileptic effects. This study also revealed an interesting discovery regarding the different intervention effects of GLS on epileptiform discharge hippocampal neurons and expression of NT-4 and N-Cadherin when GLS was added into the medium with or without Mg^2+^ ions. Further study is needed to investigate the timing and conditions of therapeutic use of GLS.
